# Machine Learning Reveals the Association Between Gene Expression and Immune Infiltration in Colorectal Cancer: A Comprehensive Study From Single‐Cell to Survival Analysis

**DOI:** 10.1111/jcmm.71049

**Published:** 2026-03-02

**Authors:** Xiaoxin Duan, Shen Huang, Yan Zhou, Tao Yang, Jiaqi Liu, Hudan Song, Pingliang Sun

**Affiliations:** ^1^ Department of Anorectal Surgery The First Affiliated Hospital of Guangxi University of Chinese Medicine Nanning Guangxi China; ^2^ Department of Anorectal Surgery The First Affiliated Hospital of Guizhou University of Chinese Medicine Guiyang Guizhou China

**Keywords:** colorectal cancer, gene expression, immune infiltration, machine learning, prognostic model, single‐cell RNA sequencing

## Abstract

Colorectal cancer (CRC) is one of the most common causes of cancer mortality globally. Analysis of immune cell infiltration patterns in the tumour microenvironment (TME) is critical to treatment outcomes, but the molecular mechanisms which regulate this process are still poorly understood. We uniquely applied machine learning to single‐cell RNA sequencing analysis to unravel the complex interaction between gene expression profiles and immune cell infiltration in CRC. We present a new computational framework that integrates different machine learning methods to analyse single‐cell RNA sequencing data from CRC patients. The system leverages unsupervised clustering, survival, and gene‐set enrichment analyses to pinpoint principal molecular signatures. CIBERSORT & ESTIMATE were used for immune cell quantification, whereas UMAP and t‐SNE were used for high‐dimensional data visualisation and pattern discovery. Our analyses uncovered gene expression signatures that closely associated with immune cell infiltration patterns in CRC. Using unsupervised clustering, we discovered two novel molecular subtypes that displayed markedly different outcomes (*p* = 0.049). We identified CD19, MAP2, CALB2 and TGFB2 as key biomarkers involved in immune modulation. However, gene enrichment analysis of these subgroups revealed new biological pathways involving the immune response. Our proposed models showed strong predictive capabilities verified by ROC curve analysis. Using single‐cell analysis to identify previously uncharacterized interactions between specific immune cell populations and tumour cells, thereby uncovering novel immune evasion mechanisms and potential immunotherapy targets within the TME. Our results uncover novel candidate biomarkers for response to immunotherapy prediction and highlight molecular profiles that could support guided treatment approaches. The predictive models derived at present have the potential to be implemented in clinical practice for decision‐making in CRC management.

## 1 Introduction

Colorectal cancer (CRC) is the third most common cause of cancer‐related death and a significant public health burden with high incidence and mortality rates. It is known that surgical techniques, systemic therapies and targeted therapy for CRC have been developed in recent years; however, the prognosis of CRC patients still remains poor, especially for advanced CRC patients [1, 2, 3]. This underscores the need for an improved understanding of the cellular and molecular mechanisms contributing to CRC, along with crosstalk that enriches the tumour microenvironment (TME) with immune cell infiltration. In particular, the former has turned out to be an important driver of cancer development and treatment response.

Until recent years, revealing the complexity of CRC has only been facilitated by advances in genomic technologies, such as next‐generation sequencing and single‐cell RNA sequencing (scRNA‐seq), enabling in‐depth dissection of the heterogeneity of CRC at unprecedented resolution. These technologies have revealed the intricate landscape of gene expression profiles and cellular component composition in CRC tumours [4, 5, 6]. Moreover, when combined with machine learning algorithms, high‐throughput data has also been instrumental in discovering novel biomarkers and designing predictive models.

One area in which it has been shown that artificial intelligence technology, and in particular machine learning, can offer a useful means of handling such complex biological data is through revealing subtle associations and patterns which more conventional analytical techniques may fail to detect. In CRC, machine learning has been employed to predict treatment responses, stratify patients, and detect molecular signatures of tumour aggressiveness and patient survival [7, 8, 9, 10].

Single‐cell RNA sequencing has changed the landscape of knowledge on the tumour heterogeneity and immune cell dynamics in the CRC microenvironment. Coupling this technology with sophisticated machine learning methods provides new views into cellular interactions and molecular networks. Indeed, recent studies have shown that the coupling of machine learning with scRNA‐seq data can be employed to identify new immune cell subtypes, phenotype their functional states, and delineate their spatial organisation within tumours. Western Single‐cell approaches have the potential to dissect complex single‐cell expression patterns and predict cell‐ cell communication networks that shape tumour progression and response to therapy using neural networks [11, 12, 13].

Different immune cells infiltrating in CRC are linked with clinical outcomes like tumour progression, response to immunotherapy and survival. Therefore, both quantitative and qualitative monitoring of the immune landscape of CRC tumours is still essential [14, 15, 16, 17]. Examples of the use of computational methods for estimating relative abundance of specific immune cell types from a mixture solely based on gene expression data offer a useful tool in the immunological toolkit without reverting to the laborious methodology of isolating and characterising each cell population (CIBERSORT and ESTIMATE).

For this study, we investigated the relationships between gene expression patterns and immune cell infiltration within the CRC tumour microenvironment using an integrated computational approach. Using machine learning algorithms and single‐cell RNA sequencing analysis, we aimed to: (1) identify gene expression signatures distinguishing different immune microenvironment states, (2) describe how specific gene expression patterns correlate with immune cell infiltration profiles, and (3) assess their combined influence on patient outcomes. Combined with the micro‐anatomical features, our approach offers the potential to establish novel frameworks in moving towards personalised and precision medicine by understanding how gene expression levels ultimately impact the immune cell diversity of CRC. This study will help to design better immunotherapy strategies and personalised treatment protocols for patients with CRC.

## 2 Methods

### 2.1 Data Acquisition

RNA expression markers have been identified through a systematic overview of that RNA expression data along with clinical data for CRC patients obtained from The Cancer Genome Atlas (TCGA) and Gene Expression Omnibus (GEO) databases [18, 19]. For RNA‐expressing data, samples with missing clinical information (survival status, tumour stage, et al.) or low‐quality sequencing data were removed. And expression values were log_2_ (FPKM + 1) transformed for normalisation. Moreover, cell transcriptional profiles that represent the granular nature of CRC were collected from GSE146771 for single‐cell RNA sequencing (scRNA‐seq) data. These preprocessed datasets were then used for clustering, survival modelling and immune infiltration characterisation.

### 2.2 Identification of CRC‐Associated Genes

Differential expression analysis was conducted in R software to compare gene expression profiles between CRC tumour and normal adjacent samples. Differentially expressed genes in CRC. We used logFC ≥ 1 to retain genes with at least a twofold change in expression and FDR ≤ 0.05 to have a threshold for false discovery. Univariate Cox regression analysis of CRC‐associated genes differentially expressed in CRC to patient survival was further conducted [17]. Using correlation analysis with clinical data, we also sought potential prognostic biomarkers. Manual balance selection is not a good option, so we wanted to studied the relationship between autophagy‐related genes and immune cell infiltration in CRC to more detail about the communication pathway of the two in the development of CRC.

### 2.3 Consensus Clustering Methodology

The ‘ConsensusClusterPlus’ R package was used for unsupervised analysis to establish the optimal number of clusters and their members according to stability. The idea is to cluster a subset of the data multiple times, which allows assessing the stability of the clusters. We used the k‐means algorithm under the framework of consensus clustering, which starts with randomly chosen cluster centroids and finds the closest cluster to each data point based on distance. Consensus plots for both items and clusters were then created in order to assess the stability of gene and cluster assignment (i.e., how well the items were grouped), which is essential for finding the appropriate number of clusters. Making consensus matrix and tree by ConsensusClusterPlus package analysis, which firstly identify two main cancer clusters: ‘CRC‐related’ and ‘non‐CRC‐related’. Survival analysis based on Kaplan–Meier was performed to compare survival between these identified clusters, so as to evaluate associativity between cluster membership and patients prognosis [20, 21]. Prior to consensus clustering, data preprocessing including L2 normalisation was performed to correct for batch effects. The high‐dimensional rendering of clustering results was most likely done by using the t‐SNE algorithm.

### 2.4 Machine Learning Approaches, Model Development and Validation

For model generalisation assessment, we partitioned the CRC dataset into TCGA and GEO cohorts, enabling cross‐source evaluation. Our training framework encompassed 10 distinct algorithms alongside 101 combinatorial approaches, specifically: random survival forests (RSF), elastic net (Enet), Lasso, ridge regression, stepwise Cox, CoxBoost, partial least squares regression for Cox models (plsRcox), super principal components (SuperPC), gradient boosting machines (GBM) and survival support vector machines (survival‐SVM). Given their superior capability in managing high‐dimensional datasets and executing feature selection, these algorithms were deemed optimal for our analysis. The selection criterion centred on achieving the highest average Harrel's concordance index (C‐index) across both cohorts, where elevated C‐index values signified superior predictive performance. Patient risk stratification followed calculation of individual risk scores using the formula: Risk score = Σ (coefficient × expression value). This computational approach enabled binary classification into high‐risk and low‐risk categories. Visualisation workflows leveraged the R package ‘ggplot2’, with Sankey diagrams specifically deployed to map risk cluster relationships. Model robustness underwent rigorous evaluation through multiple analytical approaches: Kaplan–Meier survival analysis, receiver operating characteristic (ROC) curve generation via 10‐fold cross‐validation, and decision curve analysis (DCA). These assessments spanned the complete dataset as well as TCGA‐specific and GEO‐specific subsets. The k‐fold cross‐validation methodology involved partitioning data into k segments, iteratively designating one segment as the test set while utilising the remaining k‐1 segments for training. This iterative process, executed across all possible k configurations, minimised evaluation variance and yielded more reliable performance estimates [22, 23, 24].

### 2.5 Functional Enrichment and Immune Infiltration Analysis

Using a false discovery rate (FDR) < 0.05 as the statistical significance threshold, we performed Gene Ontology (GO) classification and KEGG pathway enrichment analysis to characterise the role of DEGs in CRC. CIBERSORT and ESTIMATE algorithms in R were used to determine differences in immune cell infiltration between cohorts [25, 26].

### 2.6 Single‐Cell Data Validation

We performed comprehensive quality control and analysis of single‐cell RNA sequencing data using the ‘Seurat’ R package. Initial quality control involved filtering cells based on gene expression levels (minimum 200 features) and mitochondrial gene content (maximum 20%). The data underwent log normalisation with a scale factor of one, generating a normalised expression matrix for downstream analysis.

For dimensional reduction and visualisation, we applied Principal Component Analysis (PCA), followed by t‐SNE to display the clustering structure in two dimensions. Unsupervised clustering analysis was performed to group cells based on gene expression similarity, revealing underlying cellular heterogeneity. Cell type identification was conducted using the ‘SingleR’ package by comparing our data with reference datasets. Marker genes were identified using the ‘FindAllMarkers’ package, employing statistical methods including *t*‐tests and negative binomial generalised linear models to determine differentially expressed genes between cell populations. To address batch effects and ensure data integration quality, we implemented the Seurat integration workflow with batch correction. This analytical pipeline enabled robust characterisation of cellular heterogeneity and gene expression patterns in our CRC samples.

### 2.7 Cell–Cell Communication Analysis in Colorectal Cancer

To elucidate the intercellular signalling landscape within the CRC microenvironment, we employed the CellChat R package (version 1.5.0) to infer and visualise cell–cell communication networks based on single‐cell RNA sequencing data. Using a curated ligand–receptor interaction database, CellChat computed the probability of intercellular communication via mass action modelling, integrating both ligand and receptor expression levels. Communication strength between cell types was quantified and visualised as a weighted network, where edge width represents signalling probability and node size corresponds to the number of interactions. Subsequently, pathway‐specific analyses were performed to identify key signalling axes, including the MIF, ANNEXIN and GALECTIN pathways, which exhibited strong macrophage‐mediated activity. The resulting interaction matrices and violin plots were generated using ggplot2 for pathway‐level visualisation. All analyses were conducted with default parameters unless otherwise specified, and only communications with *p* < 0.05 and communication probability > 0.01 were considered significant.

## 3 Results

### 3.1 Differential Gene Expression in Colorectal Cancer Between Tumour and Normal Samples

**FIGURE 1:**
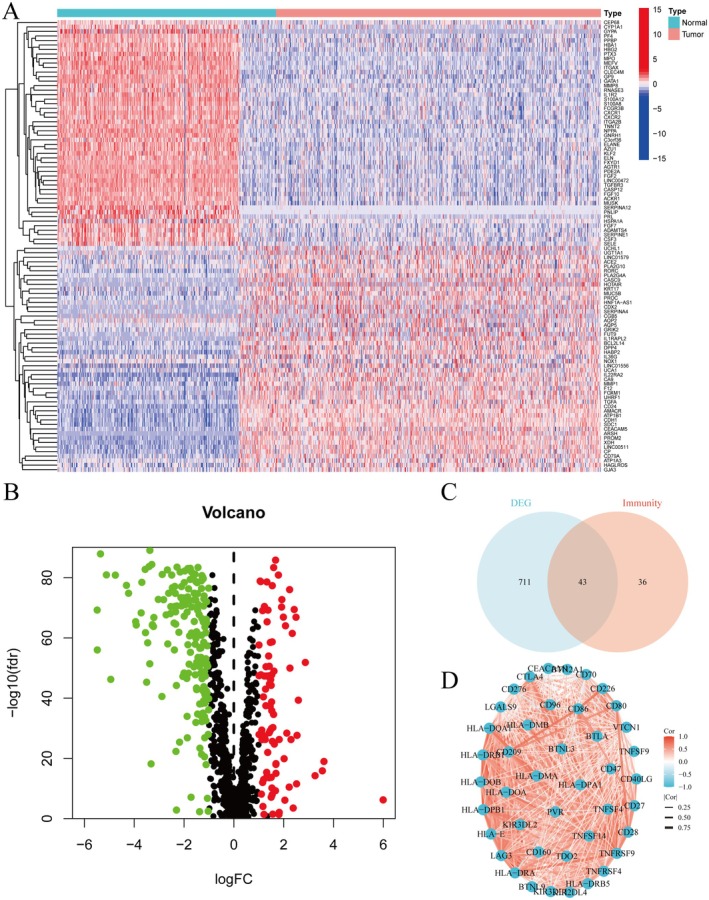
Differential gene expression in colorectal cancer between tumour and normal samples. (A) Heatmap showing differential gene expression between normal and tumour tissues in colorectal cancer. Red indicates higher expression, and blue indicates lower expression. (B) Volcano plot illustrating differentially expressed genes (DEGs). Red dots represent upregulated genes, green dots represent downregulated genes, and black dots are non‐significant. (C) Venn diagram showing overlap between DEGs and immune‐related genes. (D) Network diagram of key DEGs and their interactions. Node size indicates the degree of interaction, with colour representing expression level.

Our analysis revealed distinct gene expression patterns between tumour and normal samples (Figure 1A–D). Notably, several immune‐related genes showed significant differential expression, with 43 genes overlapping between differentially expressed and immune‐related gene sets.

### 3.2 Comprehensive Analysis of Clustering in Colorectal Cancer Samples

**FIGURE 2:**
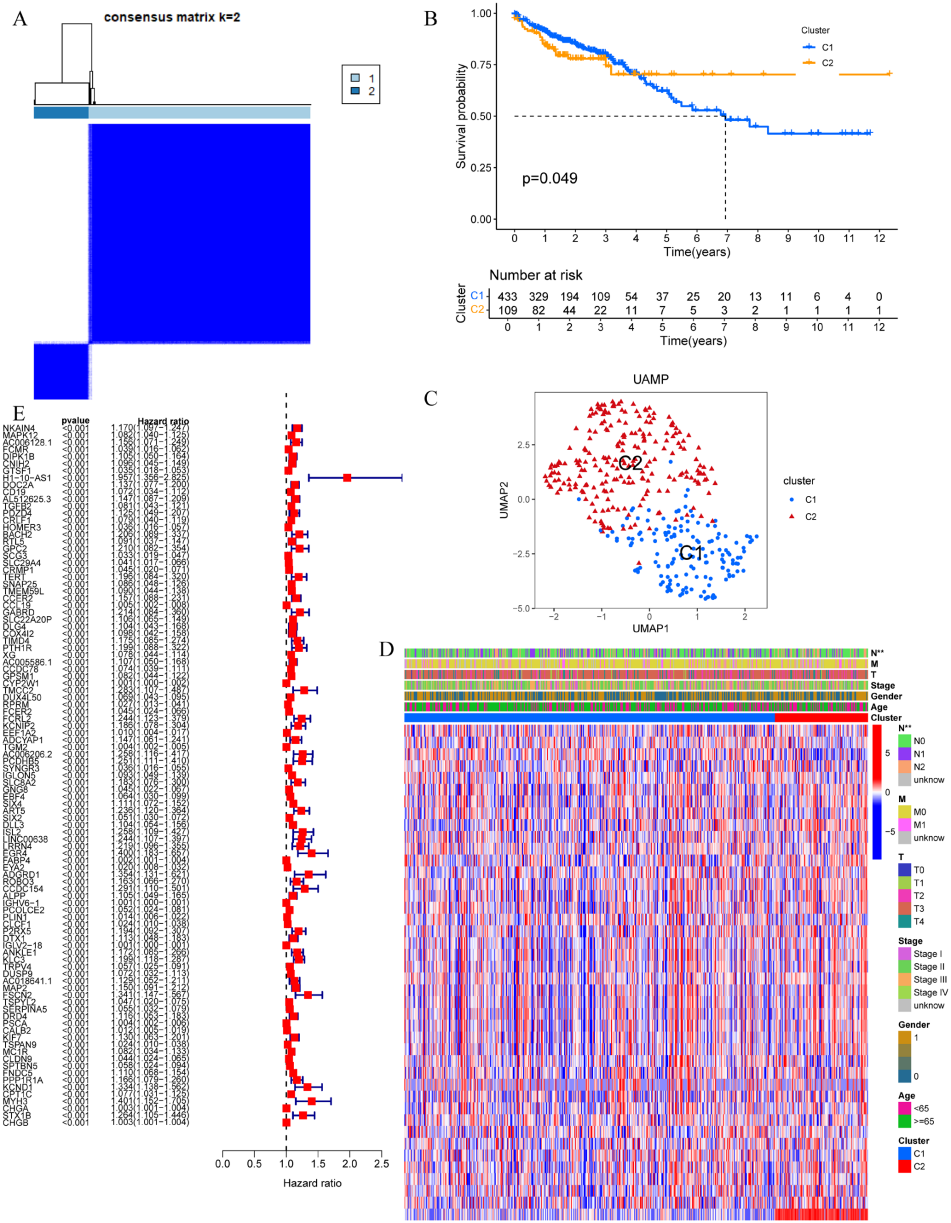
Comprehensive analysis of clustering in colorectal cancer samples. (A) Consensus matrix showing two distinct clusters in colorectal cancer samples. (B) Kaplan–Meier survival curve comparing the two clusters, with significant survival differences (*p* = 0.049). (C) UMAP plot visualising the separation of clusters C1 and C2. (D) Heatmap of gene expression profiles across samples, annotated with clinical features like stage and cluster. (E) Forest plot of hazard ratios for genes associated with survival, indicating their prognostic significance.

Figure 2A displays the consensus matrix for k = 2, indicating two distinct molecular subtypes (C1 and C2) of CRC based on differentially expressed genes (DEGs). Figure 2B presents a Kaplan–Meier survival curve comparing the two clusters, showing a significant difference in survival probability (*p* = 0.049). Figure 2C uses UMAP for dimensionality reduction, projecting high‐dimensional gene expression data into a 2D space, where two distinct molecular subtypes (C1 and C2) are clearly separated. Figure 2D is a heatmap showing gene expression patterns across samples, with annotations for clinical features such as stage and gender. Figure 2E lists hazard ratios for various genes, highlighting those significantly associated with survival outcomes.

### 3.3 Analysis of Model Performance and Gene Selection in Colorectal Cancer

**FIGURE 3:**
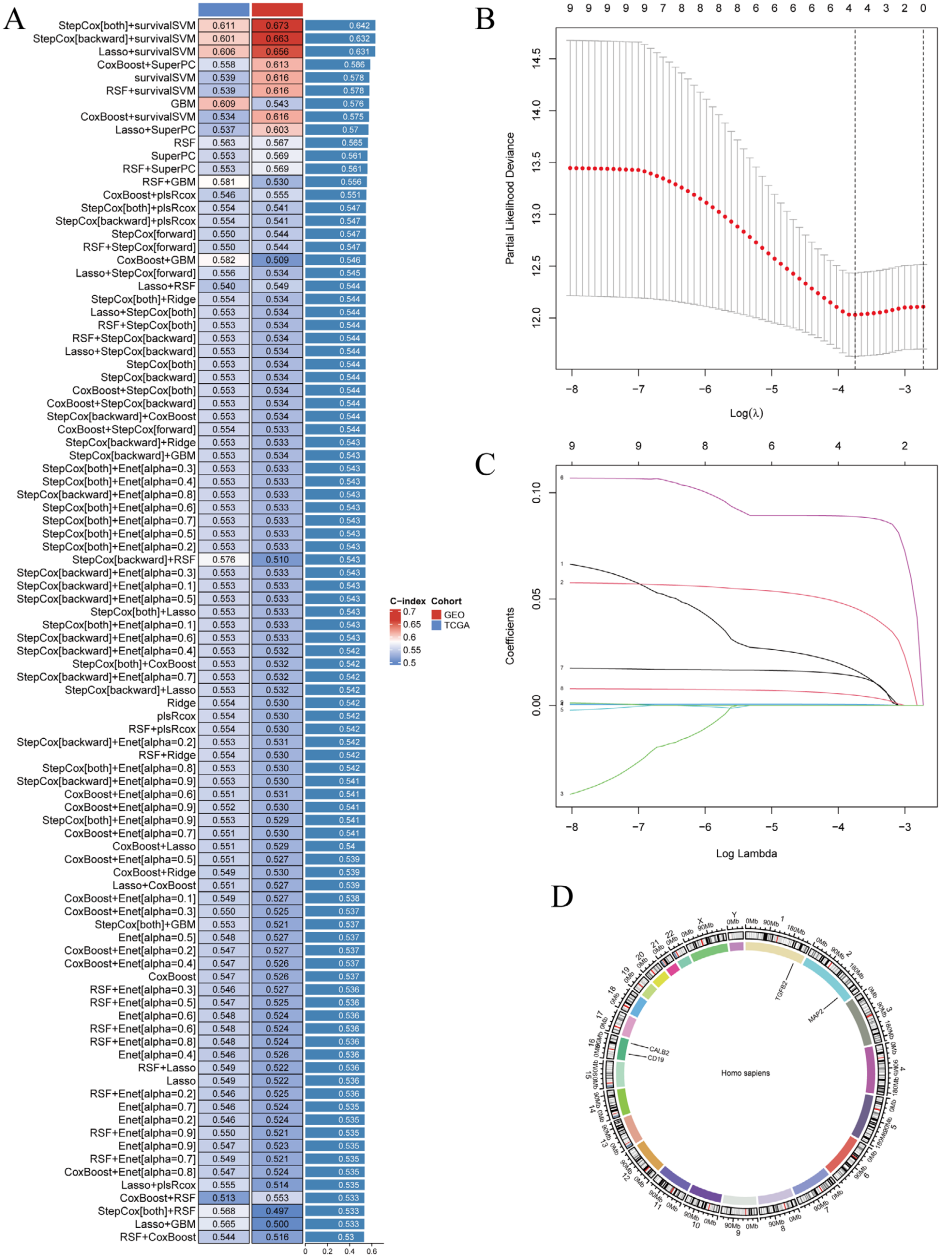
Analysis of model performance and gene selection in colorectal cancer. (A) Heatmap of C‐index values for different prognostic models, with red indicating higher performance and blue indicating lower. (B) LASSO regression plot showing the selection of optimal lambda value for feature reduction. (C) Cross‐validation plot for LASSO model, illustrating the relationship between lambda and model deviance. (D) Circular plot of key genes identified in the prognostic model, mapped onto human chromosomes.

Figure 3A presents a heatmap ranking various models based on their concordance index (C‐index), where higher C‐index values (indicating better predictive performance) are shown in red, while lower C‐index values (indicating poorer performance) are represented in blue. Figure 3B shows a plot of partial likelihood deviance against log (Lambda) from LASSO regression, identifying the optimal lambda value for feature selection. Figure 3C displays the coefficients of selected genes across different lambda values, illustrating which genes are retained or excluded as regularisation increases. Key genes retained at optimal lambda include CD19, MAP2, CALB2 and TGFB2. Figure 3D is a circos plot mapping the genomic locations of key genes identified in the analysis, highlighting their distribution across different chromosomes. The distribution pattern shows that these genes are dispersed across multiple chromosomes, suggesting potential independent regulatory mechanisms influencing CRC progression.

### 3.4 Explores Functional Enrichment Analysis in Colorectal Cancer

**FIGURE 4:**
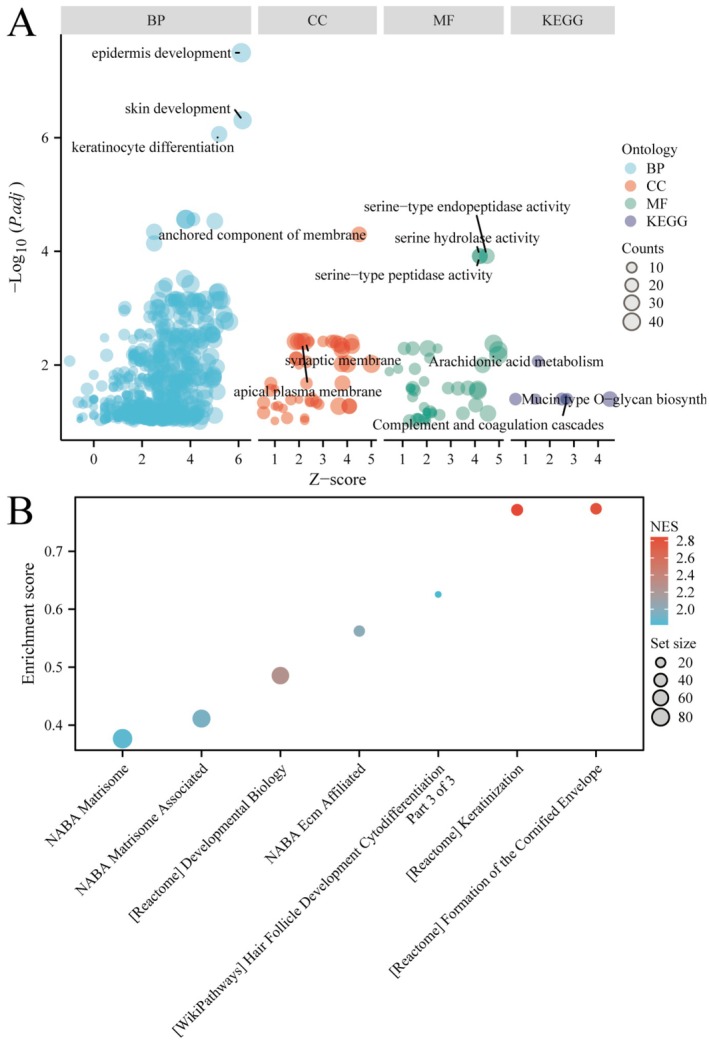
Explores functional enrichment analysis in colorectal cancer. (A) Bubble plot of gene ontology and KEGG pathway enrichment analysis. Bubbles represent biological processes (BP), cellular components (CC), molecular functions (MF) and pathways, with size indicating gene count and colour indicating significance. (B) Dot plot showing enrichment scores for key pathways, with dot size representing gene set size and colour indicating normalised enrichment score (NES).

Figure 4A is a bubble plot showing Gene Ontology (GO) and KEGG pathway enrichment. The x‐axis represents *Z*‐scores, and the y‐axis shows−log_10_ (*p*‐value). Bubbles represent biological processes (BP), cellular components (CC), molecular functions (MF) and KEGG pathways, with size indicating gene count and colour representing ontology categories. Notably, pathways related to immune response and TME remodelling were enriched, including complement and coagulation cascades, arachidonic acid metabolism and serine‐type peptidase activity. Figure 4B displays a dot plot of enriched pathways with their enrichment scores. The x‐axis lists pathways, while the y‐axis shows enrichment scores. Dot size reflects gene set size, and colour indicates normalised enrichment scores (NES), highlighting key pathways involved in the disease. Noteworthy pathways include NABA Matrisome, Matrisome‐associated, hair follicle development and keratinization and formation of the cornified envelope pathways. These findings provide molecular insights into immune infiltration and potential therapeutic targets.

### 3.5 The Prognostic Performance and Risk Factors in Colorectal Cancer

**FIGURE 5:**
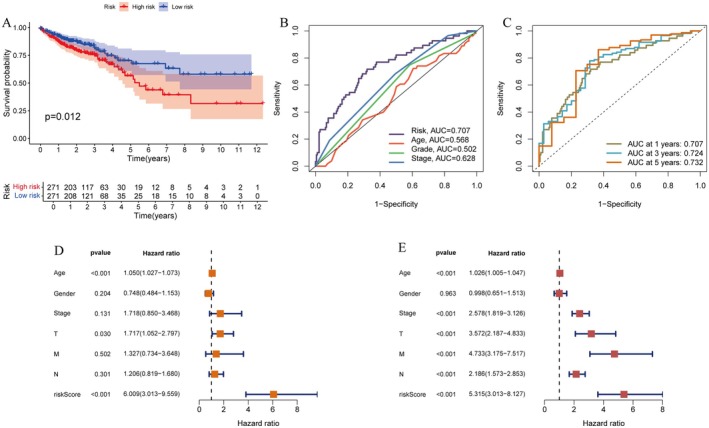
The prognostic performance and risk factors in colorectal cancer. (A) Kaplan–Meier survival curve comparing high‐risk and low‐risk groups, showing significant survival differences (*p* = 0.012). (B) ROC curves for risk score, age and stage, indicating predictive accuracy with AUC values. (C) Time‐dependent ROC curves for 1, 3 and 5‐year survival predictions. (D) Univariate Cox regression analysis forest plot showing hazard ratios for clinical variables. (E) Multivariate Cox regression analysis forest plot displaying adjusted hazard ratios for key predictors.

Figure 5A shows a Kaplan–Meier survival curve comparing high‐risk and low‐risk groups, with a significant difference in survival (*p* = 0.012). Patients in the high‐risk group exhibited a significantly poorer survival rate, while those in the low‐risk group had a better prognosis. Figure 5B presents ROC curves for risk score, age and stage, with AUC values indicating predictive accuracy: risk (0.707), age (0.568) and stage (0.628). Figure 5C displays time‐dependent ROC curves for 1‐, 3‐ and 5‐year survival predictions, with AUC values of 0.707, 0.724 and 0.732, respectively. Figure 5D provides a forest plot of univariate Cox regression analysis, showing hazard ratios and confidence intervals for factors like age, gender and stage. Figure 5E shows a forest plot of multivariate Cox regression analysis, highlighting significant prognostic factors, including stage and risk score, with their hazard ratios and confidence intervals. These findings highlight the potential clinical application of gene‐based risk scores in personalised treatment decisions by providing an independent and powerful tool for risk stratification of CRC patients, while traditional clinical factors such as tumour stage and metastasis have a significant impact on prognosis.

### 3.6 Examines Immune Cell Infiltration and Gene Expression in Colorectal Cancer

**FIGURE 6:**
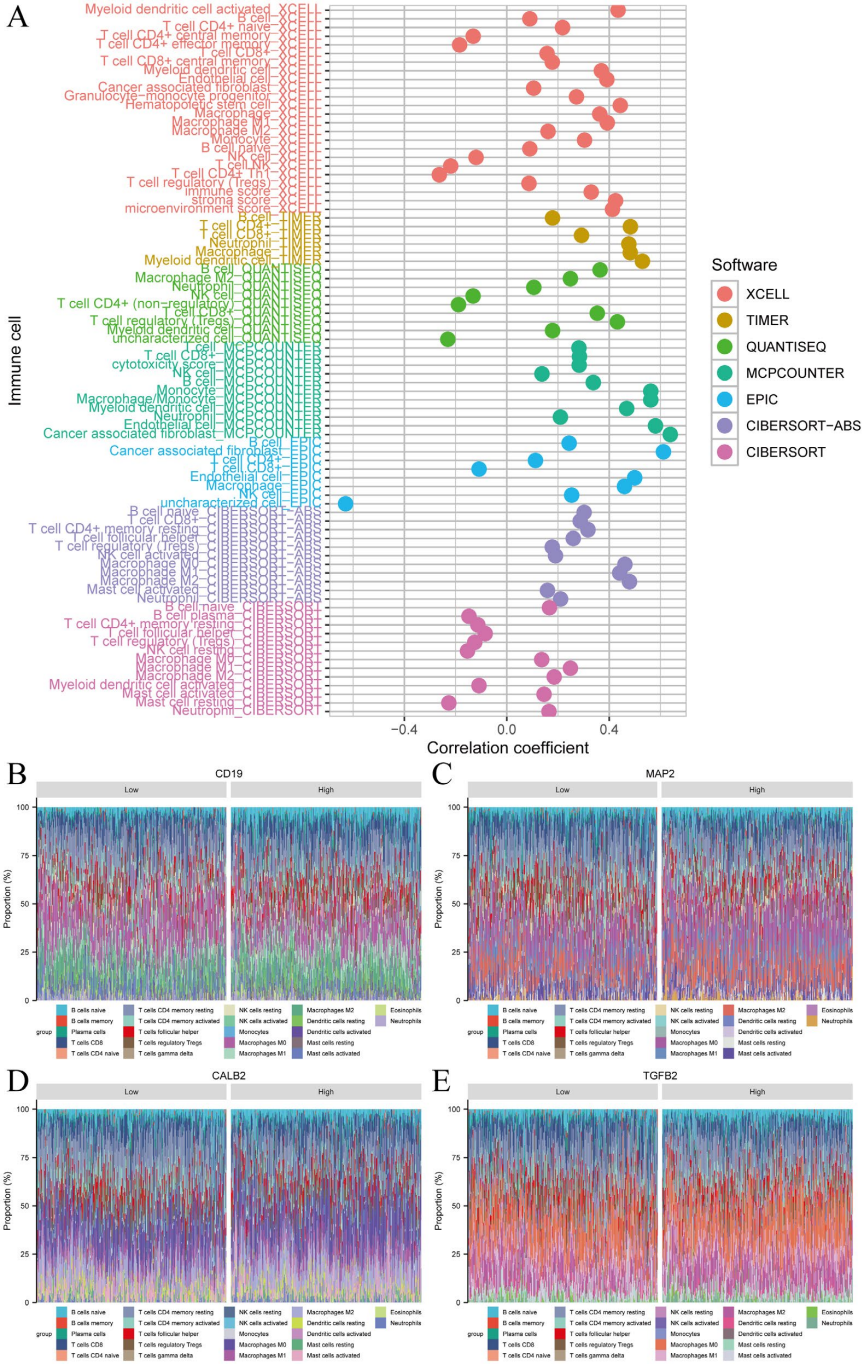
Examines immune cell infiltration and gene expression in colorectal cancer. The prognostic performance and risk factors in colorectal cancer. (A) Lollipop plot showing correlation coefficients between immune cell types and gene expression, analysed using different software tools. (B–E) Proportion plots displaying immune cell composition for low and high expression groups of CD19, MAP2, CALB2 and TGFBI, highlighting differences in immune infiltration.

Figure 6A shows the correlation between immune cell types and the combined gene expression profile of CD19, CALB2, MAP2 and TGFB2, analysed using computational tools including xCell, TIMER and CIBERSORT. The expression levels of these four genes were integrated into a comprehensive score to calculate the correlation coefficient, which was used to evaluate the immune cell infiltration patterns between different datasets. Each dot represents a correlation, with colour coding indicating the software used. Figure 6B–E display stacked bar plots of immune cell proportions associated with different gene expression levels (CD19, MAP2, CALB2 and TGFB2). Each bar shows the distribution of immune cell types in low and high expression groups, highlighting differences in immune cell infiltration patterns related to gene expression. Among them, CD19 mainly affects the infiltration of B cells, MAP2 regulates the distribution of T cells and myeloid cells, and CALB2 and TGFB2 may be involved in the establishment of an immunosuppressive environment.

### 3.7 Explores Correlations Between Gene Expression and Immune Cell Infiltration in Colorectal Cancer

**FIGURE 7:**
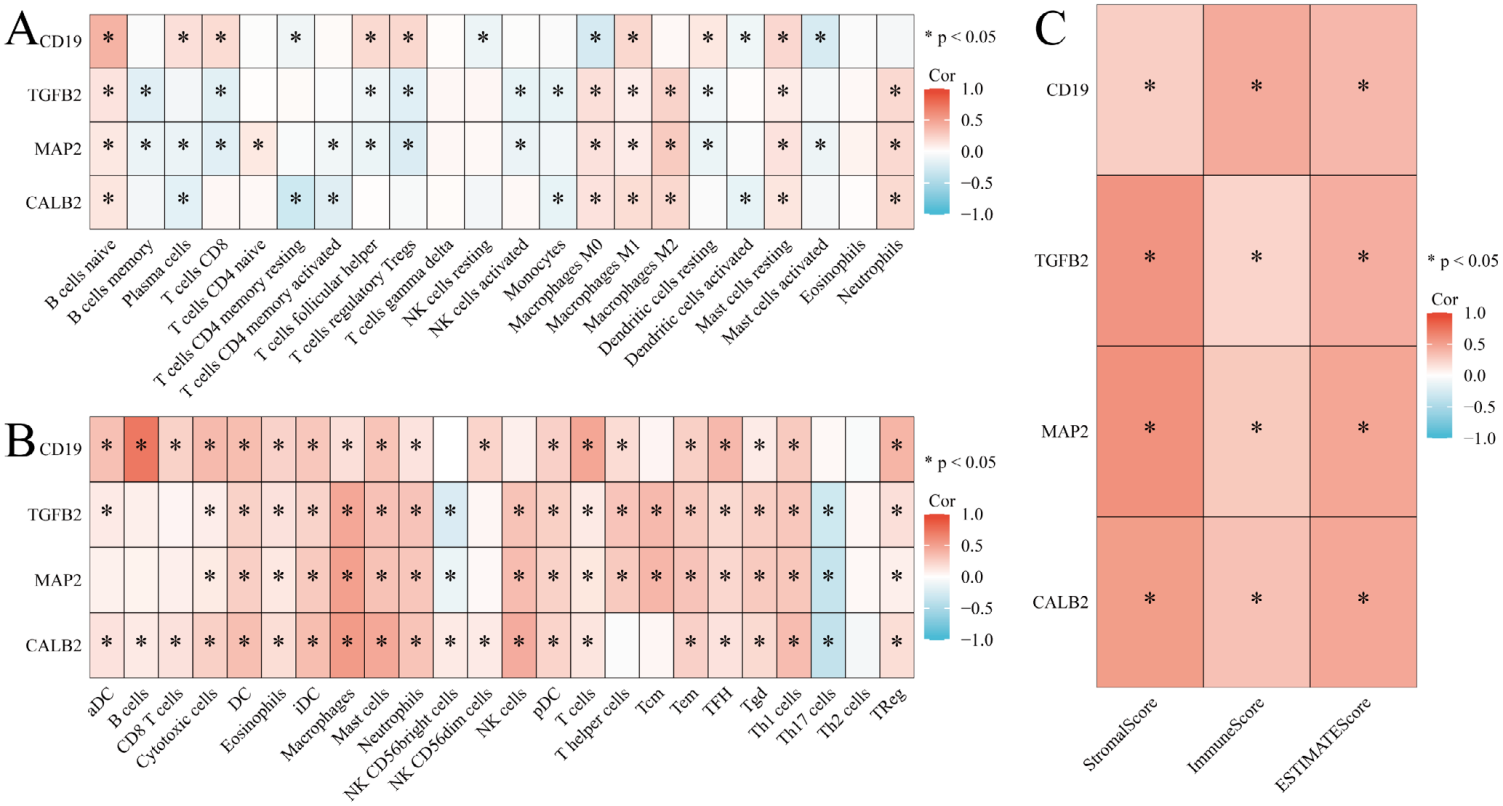
Explores correlations between gene expression and immune cell infiltration in colorectal cancer. (A, B) Correlation heatmaps showing the relationship between gene expression (CD19, TGFBI, MAP2, CALB2) and immune cell types. Red indicates positive correlation, blue indicates negative, with asterisks marking significance (*p* < 0.05). (C) Summary heatmap of significant correlations between genes and immune scores, highlighting key interactions.

Figure 7A shows a heatmap of correlation coefficients between specific genes (CD19, TGFB2, MAP2 and CALB2) and various immune cell types. Positive correlations are in red, negative in blue, with significant correlations marked by asterisks (*p* < 0.05). Figure 7B shows the correlations of four genes (CD19, TGFB2, MAP2, CALB2) with various immune cell subsets, suggesting that these genes may play important roles in immune cell function and the immune microenvironment. CD19 strongly positively correlates with B cells, suggesting a role in B cell‐mediated immune responses. TGFB2 affects M2 macrophages and regulatory T cells (Tregs), suggesting that it may affect the microenvironment by modulating immunosuppression. CALB2 is associated with B cells, dendritic cells and some T cell subsets, with a potential role in antigen presentation and inflammation. MAP2 showed heterogeneous correlations with CD8+ T cells and dendritic cells, suggesting a potential role in immune escape or suppression of the immune response. Figure 7C displays a simplified heatmap focusing on the correlation of key genes with overall immune scores, including stromal and ESTIMATE scores, emphasising significant relationships. These findings highlight the potential of these four genes as key regulators of CRC immune microenvironment, which could be leveraged as prognostic markers or therapeutic targets in immunotherapy strategies.

### 3.8 Analyzes Single‐Cell RNA Sequencing Data in Colorectal Cancer

**FIGURE 8:**
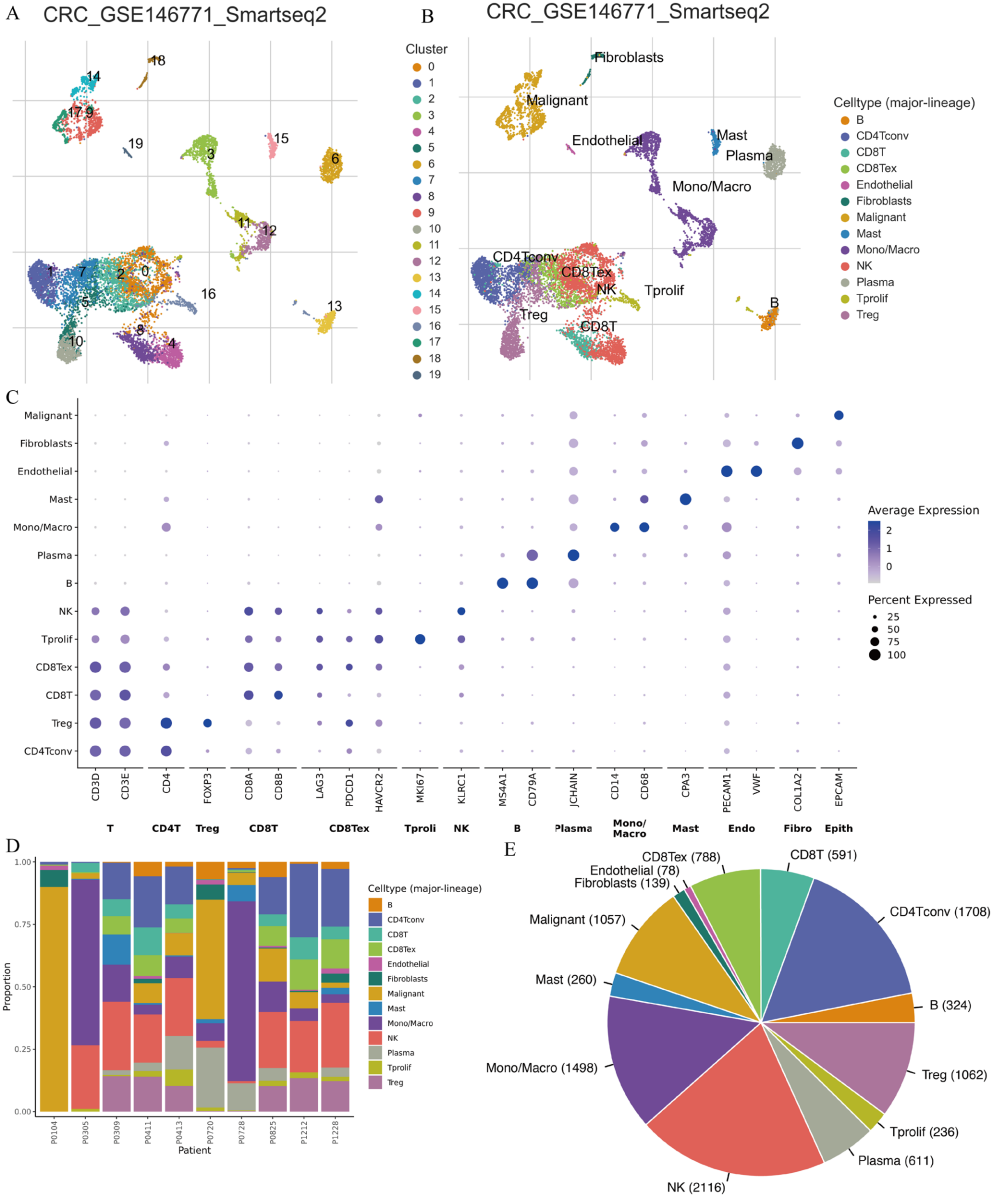
Analyzes single‐cell RNA sequencing data in colorectal cancer. (A) UMAP plot showing cell clusters from single‐cell RNA sequencing data in colorectal cancer. (B) UMAP plot with annotated cell types, including malignant, fibroblasts and immune cells. (C) Dot plot displaying average gene expression across cell types, with dot size indicating expression percentage. (D) Stacked bar chart showing cell type proportions across different patients. (E) Pie chart illustrating overall distribution of cell types in the dataset.

Figure 8A,B show single‐cell RNA sequencing (scRNA‐seq) analysis using Smart‐seq2 technology on dataset GSE146771, successfully identifying and classifying different cell types in CRC tissues, revealing significant cellular heterogeneity. The analysis identified malignant tumour cells, fibroblasts, endothelial cells and diverse immune cells, including CD4+ and CD8+ T cells, regulatory T cells (Tregs), macrophages, NK cells and plasma cells, providing high‐resolution insights into the TME. Figure 8C demonstrates cell type‐specific expression patterns of canonical marker genes,supporting the accuracy of cell populatuon annotation in the scRNA‐seq dataset. Figure 8D shows that there are significant differences in immune and stromal cell composition among different CRC patients. Some patients are enriched in Treg cells and CD8+ exhausted T cells, suggesting that immune regulation predominates in certain TMEs. Meanwhile, other patients have higher proportions of B cells, NK cells and CD4+ T cells, indicating different immune response patterns. At the same time, the differences in the distribution of fibroblasts and endothelial cells further highlight the important role of the tumour stroma in the CRC microenvironment. Figure 8E shows the overall distribution of major cell types in the CRC microenvironment. Among them, NK cells (2116) and CD4+ conventional T cells (CD4Tconv, 1708) accounted for the highest proportion, indicating that innate and adaptive immunity are jointly involved in immunoregulation in CRC. Monocytes/macrophages (Mono/Macro, 1498) also accounted for a large proportion, suggesting that they may play an important role in tumour‐associated inflammation and immunoregulation. In addition, Treg cells (1062) and CD8+ exhausted T cells (CD8Tex, 788) further revealed that an immunosuppressive microenvironment may exist in CRC. Malignant tumour cells (1057), plasma cells (611), B cells (324), mast cells (Mast, 260), proliferating T cells (Tprolif, 236), fibroblasts (Fibroblasts, 139) and endothelial cells (Endothelial, 78) together constitute a complex network of interactions between tumour stroma and immune cells. These data reveal the heterogeneity of the CRC immune microenvironment and provide a reference for considering immunotherapy strategies and microenvironment‐targeted interventions.

### 3.9 Gene Expression Across Various Cell Types in Colorectal Cancer Datasets

**FIGURE 9:**
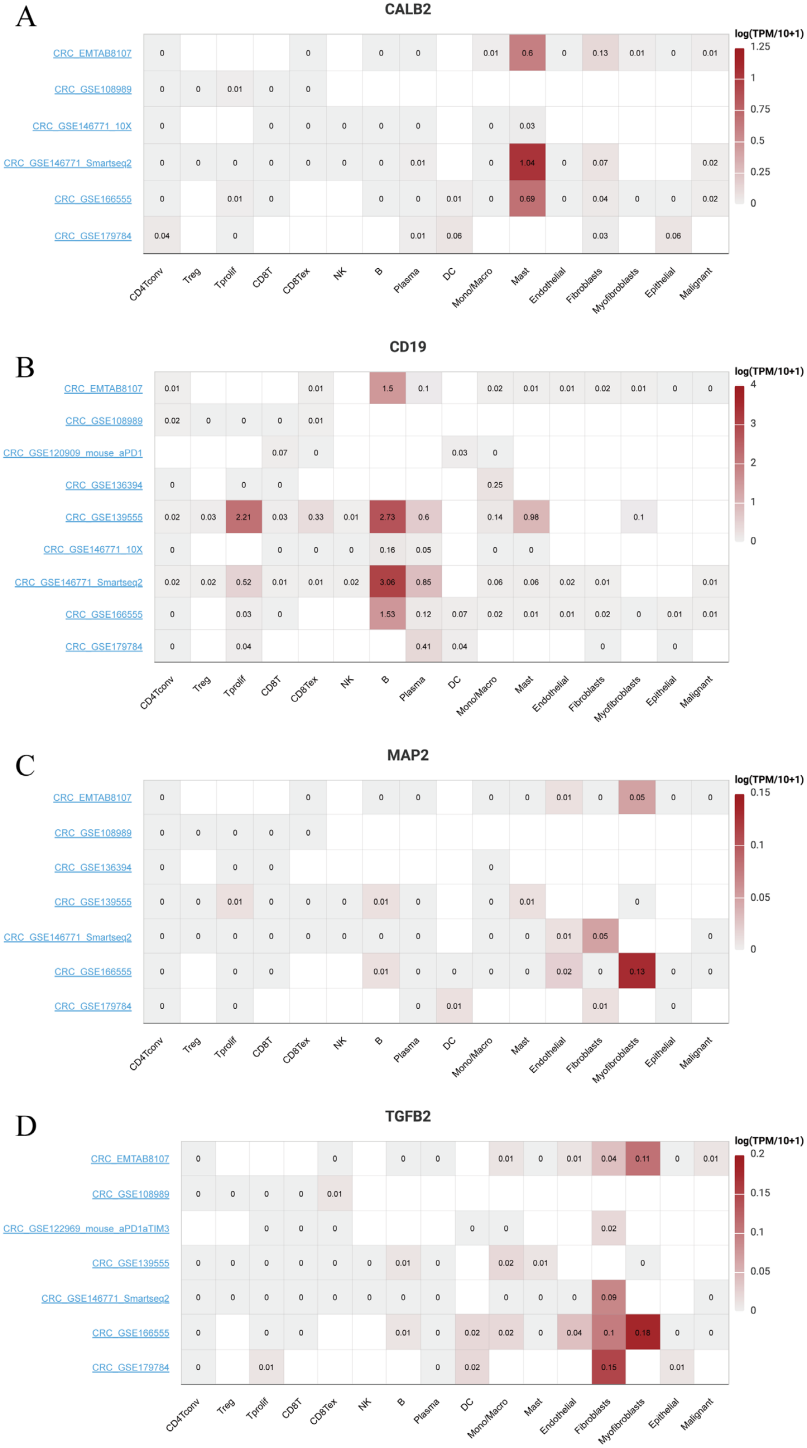
Gene expression across various cell types in colorectal cancer datasets. (A–D) Heatmaps showing expression levels of CALB2, CD19, MAP2 and TGFB2 across various cell types in different datasets. Red indicates higher expression, while white indicates lower expression, with values representing log‐transformed expression levels.

Figure 9A shows the expression of CALB2 across different immune and stromal cells. Higher expression is noted in Mast and Fibroblasts cells, indicated by darker shades of red. Figure 9B illustrates CD19 expression, with notable expression in B cells. Figure 9C displays MAP2 expression, showing significant levels in certain stromal cells, as indicated by the intensity of the colour. Figure 9D presents TGFB2 expression, with higher expression levels in fibroblasts and related cell types, marked by deeper red tones. Each heatmap uses a colour gradient to represent log‐transformed expression levels, with darker colours indicating higher expression. The datasets on the left side provide context for the expression patterns observed.

### 3.10 Gene Expression in Single‐Cell RNA Sequencing Data for Colorectal Cancer

**FIGURE 10:**
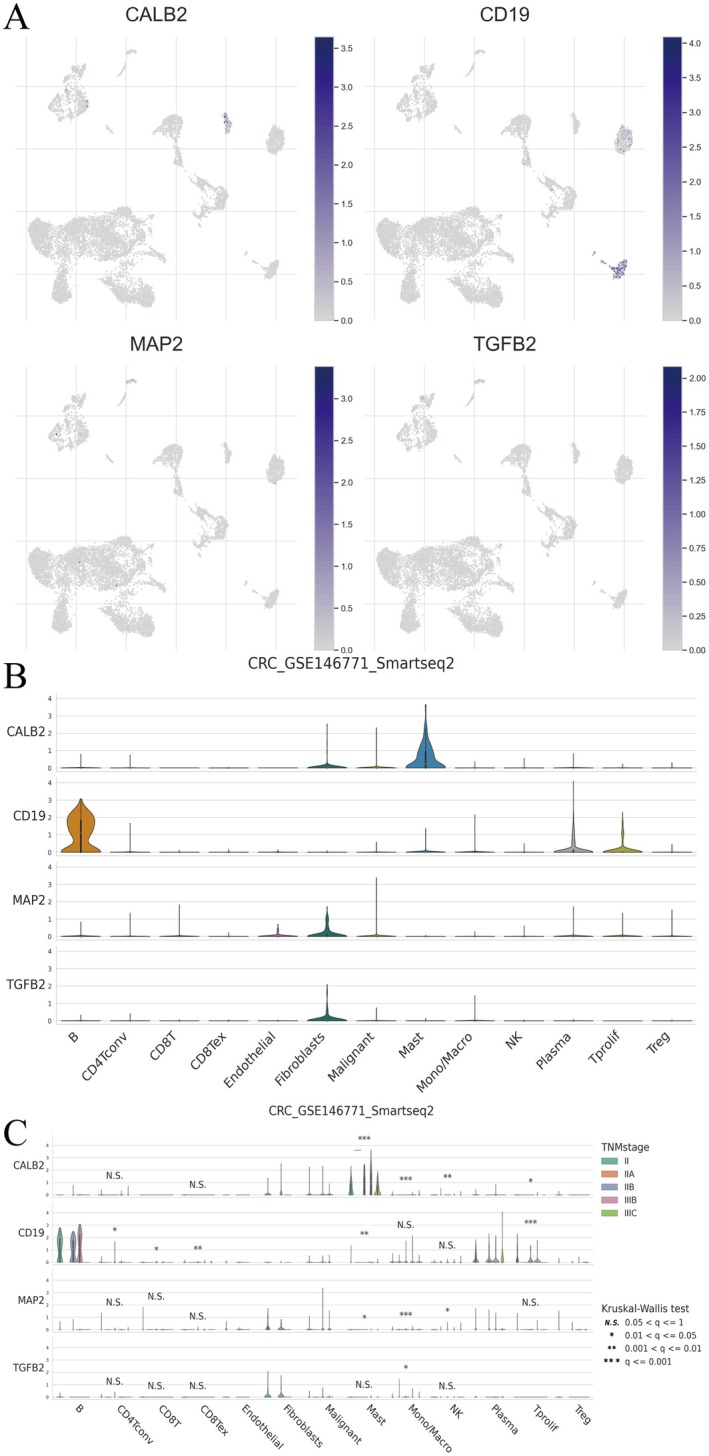
Gene expression in single‐cell RNA sequencing data for colorectal cancer. (A) Feature plots showing expression levels of CALB2, CD19, MAP2 and TGFB2 across cell clusters. (B) Violin plots illustrating gene expression distributions in different cell types. (C) Box plots comparing gene expression across tumour stages, with significance indicated by asterisks.

Figure 10A shows UMAP plots for CALB2, CD19, MAP2 and TGFB2, indicating expression across cell clusters. Darker shades of blue represent higher expression levels. Figure 10B presents violin plots displaying the distribution of gene expression across various cell types, such as CD8+ T cells, endothelial cells, fibroblasts and macrophages. Each plot highlights the variability and median expression levels within each cell type. The results showed that CD19 was significantly more expressed in B cells compared to other cell types, suggesting that CD19 may play an important role in B cell immune responses. CALB2, MAP2 and TGFB2 are mainly expressed in fibroblasts and mast cells, etc., and are suggested to be involved in the regulation of extracellular matrix and cell structure. Figure 10C provides violin plots of gene expression stratified by tumour stage, using the Kruskal–Wallis test for statistical significance. Differences in expression across stages are marked with asterisks, indicating significant variation in expression related to tumour progression. CD19 expression in different TNM stages varies greatly, suggesting that it may be associated with changes in the immune microenvironment of CRC, especially B cell infiltration. CALB2, MAP2 and TGFB2 were upregulated in some matrix‐associated cell populations and may play a role in ECM changes or matrix remodelling in the TME.

### 3.11 Intercellular Communication Reveals Dynamic Immune–Stromal Interactions in Colorectal Cancer

**FIGURE 11:**
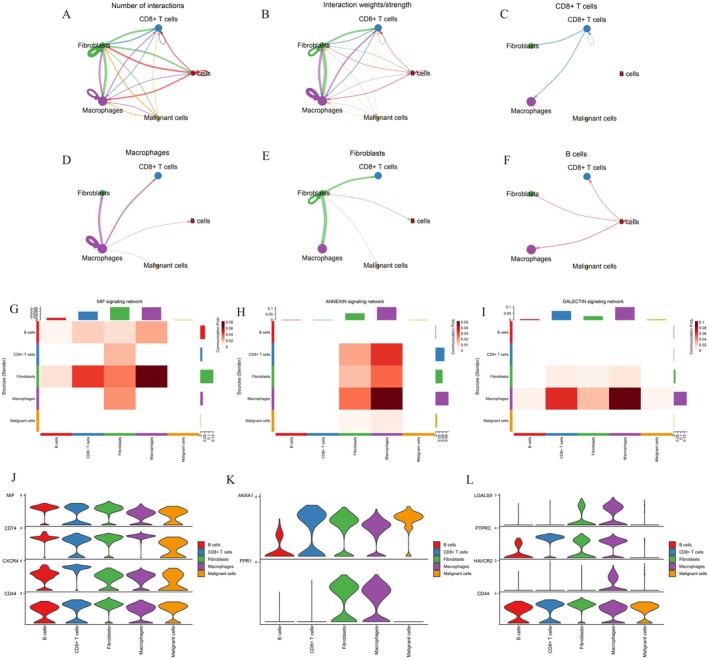
Intercellular communication patterns among major colorectal cancer cell types. (A) Shows the total number of ligand–receptor interactions, indicating strong connectivity among CD8^+^ T cells, fibroblasts and macrophages. (B) Depicts interaction strength based on communication probability, highlighting fibroblast–macrophage and CD8^+^ T cell–malignant cell communication as the most active axes. (C–F) Display cell‐type–specific communication sub‐networks. (G–I) Heatmaps illustrating communication probabilities among cell types within the MIF, ANNEXIN and GALECTIN signalling networks, respectively. The colour intensity represents the communication strength from sender to receiver. (J–L) Violin plots show the expression patterns of key ligand–receptor pairs across cell types.

Comprehensive mapping of ligand–receptor communication networks (Figure 11A–F) demonstrated that immune and stromal cells form highly interconnected networks in the CRC microenvironment. CD8+ T cells and macrophages exhibited strong bidirectional signalling with fibroblasts and malignant cells, while fibroblasts acted as major hubs coordinating multiple interaction pathways. Notably, macrophages maintained extensive connections with both malignant and immune populations, indicating their dual role in immune regulation and tumour progression. The CD8+ T cell–fibroblast interactions (Figure 11C–E) represented a prominant communication pattern, suggesting immune–matrix crosstalk that may influence cytotoxic T‐cell function and tumour invasion. Further pathway‐specific communication analysis (Figure 11G–I) identified macrophages as the main signal senders within the MIF, ANNEXIN and GALECTIN networks. The MIF–CD74–CXCR4–CD44 axis (Figure 11J) exhibited high communication probability between macrophages and malignant cells, linking pro‐inflammatory signalling to tumour proliferation. The ANNEXIN1–FPR1 pathway (Figure 11K) was enriched in fibroblast–macrophage interactions, suggesting a role in tissue remodelling and immune modulation. Meanwhile, GALECTIN (LGALS9–HAVCR2–PTPRC) signalling (Figure 11L) showed distinct cell type‐specific expression patterns, possibly mediating immune suppression through TIM‐3 activation. Collectively, these macrophage‐driven networks underscore their pivotal function as communication hubs coordinating immune and stromal responses in CRC.

## 4 Discussion

In this article, we delve into the application of machine learning and immune infiltration in the treatment of CRC, demonstrating how these technologies revolutionise our understanding of CRC's molecular mechanisms and therapeutic strategies. The application of machine learning technology in CRC research is becoming increasingly widespread, especially in handling and analysing high‐throughput data. By integrating transcriptomic data and machine learning techniques, researchers can identify features related to CRC prognosis, clinical characteristics, genomic alterations and responses to immunotherapy. For instance, the use of non‐negative matrix factorization algorithms to classify a large number of CRC samples has revealed different TME subtypes in CRC, each exhibiting unique patterns of response to immunotherapy. These findings highlight the potential of machine learning in identifying CRC subtypes and predicting treatment responses [27, 28, 29]. Additionally, machine learning models, such as random forests, support vector machines and neural networks, have been used to predict the progression and treatment response of CRC. These models can handle complex datasets and identify biomarkers associated with CRC prognosis, thereby providing possibilities for personalised therapy.

Machine learning has emerged as a powerful tool in CRC research, demonstrating remarkable versatility across multiple aspects of cancer study and treatment. In diagnostic applications, deep learning algorithms have shown exceptional performance in analysing medical imaging data, including colonoscopy images and histopathological slides, achieving high accuracy in tumour detection and classification. For molecular profiling, various machine learning approaches such as random forests and support vector machines have successfully identified gene signatures and molecular subtypes, leading to improved patient stratification [30, 31, 32].

Immune infiltration plays a crucial role in the treatment of CRC. By analysing the composition of immune cells in the TME, researchers can assess patients' responses to immunotherapy. For example, algorithms like CIBERSORT and ESTIMATE have been used to evaluate the levels of immune cell infiltration in CRC samples, revealing different compositions of immune cells in different CRC subtypes. This information is vital for developing therapeutic strategies targeting specific immune cell types [33, 34, 35, 36]. The application of single‐cell RNA sequencing (scRNA‐seq) technology further enhances our understanding of the heterogeneity of immune cells in CRC. Recent advances in single‐cell RNA sequencing have revolutionised our understanding of CRC heterogeneity. This technology has enabled the detailed characterisation of distinct cell populations within the TME, revealing previously unknown cellular states and developmental trajectories. Key findings include the identification of novel tumour cell subpopulations with varying degrees of stemness and metastatic potential, the discovery of specific fibroblast subtypes that contribute to cancer progression, and the detailed mapping of immune cell dynamics during tumour evolution. Single‐cell analysis has also uncovered new mechanisms of drug resistance and immune evasion, highlighting potential therapeutic targets [37, 38].

Our study, through the comprehensive analysis of differential gene expression between CRC tumour and normal samples, reveals key gene changes during tumorigenesis. Using heatmaps and volcano plots, we identified hundreds of genes that are differentially expressed in CRC, whose expression changes may be closely related to tumour development and patient prognosis. Notably, we observed an overlap with immune‐related genes, indicating that the immune response may play an important role in CRC. Further cluster analysis and survival analysis revealed two distinct CRC subtypes with significant differences in survival probabilities, a finding that may aid in the future precise stratification of CRC patients for treatment. Through the performance evaluation and gene selection of machine learning models, we identified multiple genes closely related to CRC prognosis, which are CD19, MAP2, CALB2 and TGFB2, and constructed a prognostic model to predict the survival risk of patients. Functional enrichment analysis further revealed the biological processes and signalling pathways involved in these differentially expressed genes, providing a new perspective for understanding the molecular mechanisms of CRC. Additionally, our study explored the relationship between immune cell infiltration and gene expression, finding that specific gene expression patterns are associated with the distribution of immune cell types, which may be significant for the development of new immunotherapy strategies. Finally, through the analysis of single‐cell RNA sequencing data, we further revealed the heterogeneity of gene expression across different cell types in the CRC tumour microenvironment.

We found that CD19, MAP2, CALB2 and TGFB2 were identified as key regulators of the immune microenvironment in CRC. CD19 was strongly associated with B cell infiltration, suggesting its role in modulating B cell‐mediated immune responses. CD19^+^ B cells have been found to influence tumour progression, with recent studies highlighting their potential role in the TME of CRC [39]. MAP2 showed heterogeneous correlations with CD8+ T cells and dendritic cells, indicating its potential involvement in immune escape mechanisms or tumour immune evasion. MAP2, primarily known for its role in stabilising microtubules in neurons, has been increasingly recognised for its involvement in tumorigenesis [40]. CALB2 was primarily expressed in B cells, dendritic cells and certain T cell subsets, implying its function in antigen presentation and immune regulation. Functional enrichment analysis suggests that CALB2 may serve as a biomarker for early‐stage CRC, making it a potential target for prognostic evaluations [41]. TGFB2 showed a strong correlation with M2 macrophages and regulatory T cells (Tregs), highlighting its role in promoting an immunosuppressive microenvironment. TGFB2 is a well‐established immunosuppressive factor in CRC. Studies have shown that mutations in the TGF‐beta pathway were more common in older CRC patients and colon primaries [42]. This data not only enhances our understanding of the interactions between CRC tumour cells and immune cells but also may identify new therapeutic targets, providing possibilities for personalised medicine. Overall, our study results emphasise the importance of gene expression analysis and immune cell infiltration in the prognosis assessment of CRC and provide new directions for future therapeutic research.

By integrating machine learning and immune infiltration analysis, researchers can gain a more comprehensive understanding of the complexity of CRC and develop more effective treatment strategies. For example, immune‐related genes and pathways identified by machine learning models can serve as potential targets for immunotherapy. At the same time, by analysing patterns of immune cell infiltration, it is possible to predict patients' responses to specific immunotherapies, thus achieving precision medicine.

### 4.1 Limitations

Methodological limitations also warrant consideration. The interpretability of our machine learning models remains a challenge, as the complex nature of these algorithms often makes it difficult to fully understand the decision‐making process. While our bioinformatics analyses have yielded promising results, these computational findings require further experimental validation to confirm their biological significance. Furthermore, the depth and coverage of our single‐cell sequencing data may not be sufficient to capture the full complexity of cellular heterogeneity within the TME.

## 5 Conclusion

In conclusion, this study successfully identified key prognostic genes and immune microenvironment characteristics in CRC by integrating machine learning and immune infiltration analysis methods, leading to the construction of a valuable prognostic model. These findings not only deepen our understanding of the molecular mechanisms of CRC but also provide new insights for clinical practice.

## Author Contributions

Xiaoxin Duan likely conducted primary research, data analysis and drafted the manuscript. Shen Huang and Yan Zhou contributed to data collection, statistical analysis or experimental procedures. Jiaqi Liu contributed to data collection, statistical analysis or experimental procedures. Hudan Song contributed to data collection, statistical analysis or experimental procedures. Tao Yang provided clinical samples or pathological expertise. Pingliang Sun Senior researcher who designed the study, supervised the research, and takes academic responsibility for the work. All authors have read and approved the final manuscript.

## Funding

This work was supported by the following grants: the National Natural Science Foundation of China (Regional Science Fund Project) (Grant No. 82160906); the ‘Guipai Xinglin’ Elite Talent Program of Guangxi University of Chinese Medicine (Grant No. 2022C021).

## Ethics Statement

The authors have nothing to report.

## Consent

All authors reviewed and approved the final manuscript.

## Conflicts of Interest

The authors declare no conflicts of interest.

## Data Availability

For further information, please contact the corresponding author.
